# RRx-001 followed by platinum plus etoposide in patients with previously treated small-cell lung cancer

**DOI:** 10.1038/s41416-019-0504-8

**Published:** 2019-06-24

**Authors:** Daniel Morgensztern, Michal Rose, Saiama N. Waqar, John Morris, Patrick C. Ma, Thomas Reid, Christina E. Brzezniak, Karen G. Zeman, Arvinda Padmanabhan, JoAnn Hirth, Alexander I. Spira, Jane B Trepel, Sukhmani K. Padda

**Affiliations:** 1 Washington University School of Medicine, St. Louis, MO USA; 2 VA Connecticut, West Haven, CT USA; 3 University of Cincinnati Cancer Institute, Cincinnati, OH USA; 4 West Virginia University, Morgantown, WV USA; 5 Memorial Hospital, South Bend, IN USA; 6 Walter Reed National Military Medical Center, Bethesda, MD USA; 7 Baptist Health, Lexington, KY USA; 8 Henry Ford Allegiance Health, Jackson, MI USA; 9 Virginia Cancer Specialists, Fairfax, VA USA; 10 National Cancer Institute, Bethesda, MD USA; 11 Stanford University School of Medicine, Stanford, CA USA

**Keywords:** Cancer, Small-cell lung cancer, Cancer therapy, Immunotherapy

## Abstract

**Background:**

This exploratory single-arm phase II study evaluated the efficacy and safety of RRx-001 followed by reintroduction of platinum plus etoposide in patients with previously treated small-cell lung cancer (SCLC).

**Methods:**

Patients were treated with RRx-001 4 mg IV on day 1 of each week of a 21-day cycle followed at progression by re-challenge with etoposide 80–100 IV mg/m^2^ on days 1, 2 and 3 and cisplatin 60–80 mg/m^2^ IV on day 1 or carboplatin AUC 5–6 IV on day 1, every 21 days. The primary end points were overall survival (OS) and overall response rate to platinum regimen.

**Results:**

Twenty-six patients were enroled and received at least one dose of RRx-001. The median number of prior lines of therapy was 2 (range 1–9) and 19 (73.1%) patients had platinum-resistant disease. In the intention-to-treat population, one patient (3.8%) had complete response and six (23.1%) had partial response on platinum plus etoposide. The estimated median and 12-month OS from enrolment were 8.6 months and 44.1%, respectively. The most common treatment-emergent adverse event from RRx-001 was mild discomfort at the infusion site (23%).

**Conclusions:**

RRx-001 followed by re-challenge with platinum plus etoposide chemotherapy is feasible and associated with promising results.

**Clinical trial registration:**

NCT02489903.

## Background

Small-cell lung cancer (SCLC) accounts for approximately 13% of all cases of lung cancer in the United States and is characterised by rapid growth and early development of metastatic disease.^1^ Despite excellent response to first-line platinum-based chemotherapy regimens, the majority of patients with limited-stage disease and virtually all patients with extensive-stage disease develop tumour progression.^2^ The strongest predictor for outcomes in patients with tumour progression after first-line chemotherapy in SCLC is the duration of response. Outcomes in subsequent lines of therapy, including response rates and survival, are better for patients with platinum-sensitive disease, defined as relapse more than 3 months from completion of initial therapy, than in those with platinum-refractory or resistant disease, defined as progression during initial therapy or relapse within 3 months from completion of the initial therapy. Patients with tumour relapse 6 or more months after completion of first-line therapy, may benefit from repeating treatment with the original platinum-based regimen.^3–5^ Although the addition of atezolizumab to platinum and etoposide was associated with a modest improvement in overall survival (OS) compared with chemotherapy alone in the first-line setting, there are no data on the use of this regimen in previously treated patients.^6^

Topotecan is the only approved drug in the second-line setting for SCLC.^7^ Among patients treated with topotecan in a large randomized clinical trial, the response rates, median progression-free survival (PFS) and OS were 23.1%, 4.3 months and 9 months, respectively, in those with chemotherapy-sensitive disease and 9.4%, 2.6 months and 5.7 months, respectively, in those with chemotherapy-resistant disease. Nivolumab, a monoclonal antibody against programmed death receptor-1 (PD-1), was approved by the Food and Drug Administration for patients with SCLC progressing after platinum-based chemotherapy and at least one other line of therapy, based on the results of the CheckMate-032 study, where, among the 98 patients enroled, the response rate, median PFS and 1-year PFS were 10%, 1.4 months and 11%, respectively.^8^ The overall lack of survival improvement for SCLC over the past three decades indicates a clear need for novel treatment approaches.^9,10^

RRx-001 is a dinitroazetidine derivative that, once injected intravenously, rapidly penetrates red blood cells (RBCs), where it binds to the haemoglobin β-chain cysteine 93 residue (Cys-β^93^), forming stable adducts, and reduces gluthatione.^11,12^ RRx-001 is associated with pleiotropic effects, including upregulation of oxidative stress, epigenetic modulation and macrophage polarisation to M1 phenotype. In the phase 1 study, which included 25 patients with advanced-stage solid tumours, RRx-001 was well tolerated over six dose cohorts ranging from 10 to 83 mg/m^2^ administered intravenously once or twice per week, with no dose-limiting toxicities and no treatment-related deaths.^13^ The most common adverse events in the phase 1 study were pain at the infusion site and arm swelling, which occurred in 21 patients (84%) and 8 patients (32%), respectively. Due to the epigenetic and immunologic effects, we postulated that RRx-001 may re-sensitise the tumours to platinum-based chemotherapy.

QUADRUPLE THREAT is an exploratory open-label phase 2 study evaluating the use of RRx-001 priming followed by re-challenge with platinum-based chemotherapy in patients with previously treated SCLC, *EGFR* mutated non-small-cell lung cancer (NSCLC), high-grade neuroendocrine tumours and ovarian tumours. Here, we report the initial cohort of patients with SCLC.

## Methods

### Patients

Patients 18 years or older with histologically or cytologically confirmed small-cell lung carcinoma, previously treated with a platinum-based chemotherapy regimen, were eligible if they had tumour relapse within 6 months from completion of the last cycle of first-line chemotherapy and/or received two or more lines of prior therapy. Other key eligibility criteria included radiologically measurable disease by Response Evaluation Criteria in Solid Tumour (RECIST) 1.1,^14^ Eastern Cooperative Oncology Group (ECOG) performance status 0 to 2, adequate haematologic, renal and hepatic function, and no other current active malignancy requiring anticancer therapy. Patients with symptomatic brain metastases, history of severe hypersensitivity to a platinum drug or use of platinum therapy on more than three separate lines of therapy for advanced disease were excluded from the study. Tumour progression within 3 months from the last cycle of first-line platinum-based chemotherapy was classified as platinum-resistant disease, whereas progression more than 3 months from completion of first-line therapy was classified as platinum-sensitive disease.

The study was approved by the Institutional Review Board at each of the participating sites and conducted in accordance with the principles of good clinical practice and the declaration of Helsinki. All patients provided written informed consent prior to treatment initiation.

### Treatment plan

Patients received 4 mg of RRx-001, mixed and co-infused intravenously with 12ml of their own blood over 10–30 minutes once weekly. The dose of 4 mg was chosen due to the lack of dose-related efficacy and feasibility of administration, since with a 1 to 5 dilution of drug to blood only 10 ml of blood would be required for 4 mg whereas a 10 mg dose would require 50 ml of blood, which could increase the risk of clotting and haemolysis prior to the infusion. The co-infusion was initially performed through an intravenous bag, which was subsequently changed to an infusion device after a protocol amendment. The bagless device comprised a multi-position stopcock to which three syringes were attached, intravenous tubing, a filter and a needle for removal of blood. With this approach, the infusion time was decreased from approximately 60 minutes to approximately 15 minutes. Premedication was administered within 10–50 minutes prior to each RRx-001 infusion and included dexamethasone 10 mg, administered either orally or intravenously, and acetaminophen 500 mg or aspirin 81 mg, administered orally.

Treatment with RRx-001 was continued until the development of tumour’s radiologic or clinical progression or unacceptable toxicity, with each cycle defined as three weekly treatments. Contrast-enhanced computed tomography scans were initially performed every 12 weeks. Nevertheless, the imaging frequency was changed to every 6 weeks or earlier according to the investigator’s discretion, after a protocol amendment for both RRx-001 priming and platinum re-introduction. At the time of tumour progression or clinical deterioration on RRx-001, patients started a re-challenge with etoposide 80–100 mg/m^2^ on days 1 to 3 and either cisplatin 60–80 mg/m^2^ on day 1 or carboplatin AUC 5–6 on day 1 for up to a total of six cycles, followed by close observation. Dose reductions of RRx-001 to 2 mg (first reduction) and 0.5 mg (second reduction) were allowed in case of infusion reactions and any grade 3 or 4 drug-related toxicity per the investigator’s discretion. Dose modifications of the platinum doublets were performed according to institutional guidelines and at the discretion of the investigator. Adverse events (AEs) were categorised and graded according to the National Cancer Institute Common Terminology Criteria for Adverse Events (NCI-CTCAE) version 4.03.

The primary end points were OS, defined as the time from study enrolment until death from any cause, and overall response rate (ORR) to platinum plus etoposide, defined as the proportion of patients that achieved a best overall response of complete response (CR) or partial response (PR) by RECIST 1.1 criteria, both in the intention-to-treat population (ITT). Confirmation of responses was not required for the primary end point. Secondary end points included PFS, defined as the time from study enrolment to tumour progression on platinum plus etoposide or death (whichever comes earlier), PFS1 and PFS2, defined as progressive disease or death on RRx-001 and platinum plus etoposide, respectively, disease control rate (DCR) for RRx-001 and chemotherapy, defined as CR, PR or stable disease (SD) and toxicity. Time-to-event end points were summarised using the Kaplan−Meier method, with patients who were lost to follow-up censored at the time of the last contact. Overall response rate was summarised by the number and percent of patients with partial or complete response, along with the 95% confidence interval (CI) based on the Clopper−Pearson method. All analyses were performed in R statistical software package version 3.5 (R Core Team, 2018).

With the target ORR of 20% or higher, which was considered clinically meaningful and worthy of further evaluation based on the patient population meeting the eligibility criteria, the initial part of the study was designed to accrue 26 patients based on an 80% CI and 10% precision. Since the study was considered exploratory and hypothesis generating, the sample size was not based on power considerations.

## Results

### Patient characteristics

Between December 2015 and May 2018, 26 patients with previously treated SCLC were enroled and received at least one dose of RRx-001. The median age was 62 years (range 39–83), most patients were males (54%) and 77% had a baseline ECOG performance score of 1 (Table 1). All patients had been previously treated with etoposide plus platinum, either carboplatin or cisplatin. Eleven patients (42%) were treated with platinum-based chemotherapy as their last line of therapy prior to enrolment. Eight patients (31%) received more than one course of platinum-based chemotherapy. Nineteen patients (73.1%) were resistant to first-line chemotherapy and the median number of prior lines of therapy was 2 (range 1–9).

**Table 1:** Baseline characteristics of 26 patients

Median age years (range)	62 (39–83)
*Sex*	
Male	14 (53.8%)
Female	12 (46.2%)
*Race*	
White	19 (73.2%)
Black	5 (19.2%)
Other	2 (7.6%)
*ECOG performance status*	
0	4 (15.4%)
1	20 (76.9%)
2	2 (7.7 %)
*Sensitivity to first-line chemotherapy*	
Sensitive	7 (26.9%)
Resistant	19 (73.1%)
*Number of prior lines of therapy*	
1	9 (34.6%)
2	8 (30.8%)
≥3	9 (34.6%)

### Treatment exposure

The median number of RRx-001 doses prior to starting platinum plus etoposide was 7.8 (range 3–24). No patients required dose reduction for RRx-001 and radiological or clinical progression was the reason for treatment discontinuation in all cases. The median number of cycles of platinum plus etoposide was three (range 0–6). The reasons for treatment discontinuation of platinum-doublet chemotherapy included progressive disease (PD), fatigue, weight loss, dysphagia, hypotension, depression and intracranial bleeding from known brain metastasis.

### Efficacy

Among the 26 patients enroled into the study, 23 were evaluable for radiographic responses to RRx-001 and 19 were evaluable for platinum plus etoposide. There were three patients not evaluable for response to RRx-001 and seven for platinum plus etoposide, including the three patients who discontinued therapy prior to starting the chemotherapy, due to non-compliance, rapid progression or discontinuation of the treatment before repeated imaging could be performed.

**Fig. 1:**
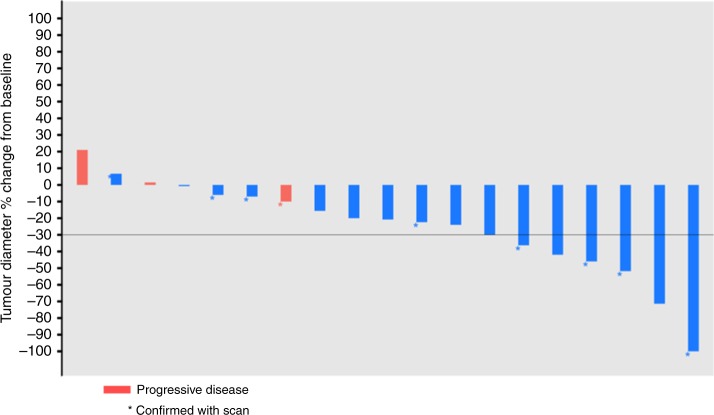
Best overall response to rechallenge with platinum plus etoposide

In the ITT analysis, one patient achieved PR (3.8%, 95% confidence interval [CI] 0.1−19.6%), seven patients achieved SD (26.9%, 95% CI 11.6–47.8%) and fifteen patients had PD (57.7%, 95% CI 36.9–76.6%) as the best response during treatment with RRx-001. Response rates to subsequent platinum plus etoposide in the ITT analysis included one CR (3.8%, 95% CI 0.1–19.6%) and six PRs (23.1%, 95% CI 8.9–43.3%), for an ORR of 26.9% (95% CI 11.6–47.8%). Nine patients achieved SD (34.6%, 95% CI 17.2–55.7%) and three patients had PD (11.5%, 95% CI 2.4–25.1%). The best overall responses to platinum plus etoposide are described in Fig. 1.

**Fig. 2:**
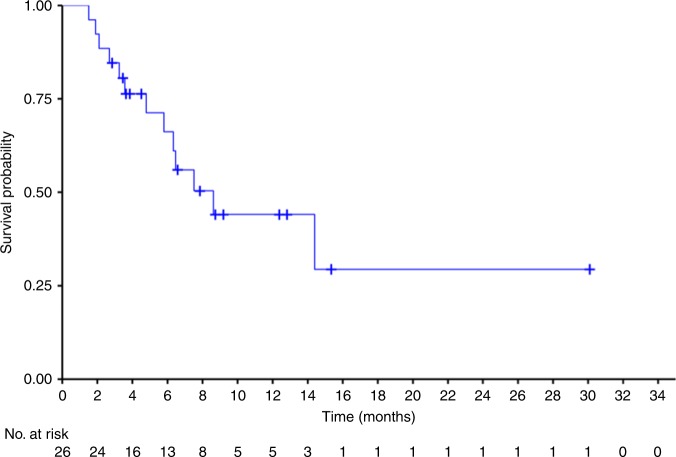
Kaplan−Meier estimates for overall survival

With a median follow-up of 7.3 months (range 1.5–30.1 months), the median OS was 8.6 months (95% CI 5.8−not reached [NR]) (Fig. 2). The estimated 6-month and 12-month OS was 66.2% and 44.1%, respectively.

**Fig. 3:**
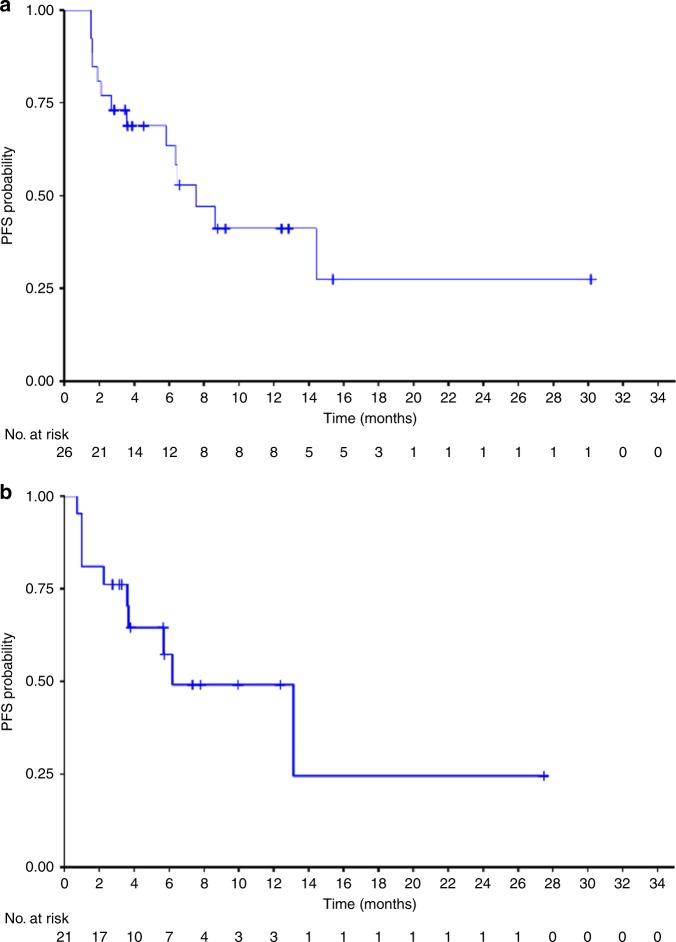
**a** Kaplan–Meier estimates for progression-free survival from starting RRx-001. **b** Kaplan–Meier estimates for progression-free survival from starting platinum plus etoposide

**Fig. 4:**
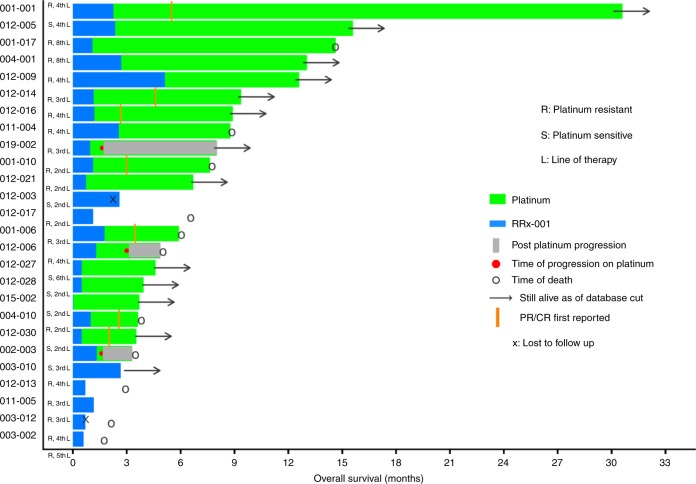
Duration of benefit

The median PFS from the first dose of RRx-001 to trial discontinuation due to clinical or radiologic-based progressive disease on platinum plus etoposide or death was 7.5 months (95% CI: 5.8–NR) (Fig. 3a), whereas the median PFS from platinum plus etoposide (PFS2) was 6.2 months (95% CI: 3.7–NR) (Fig. 3b). The median PFS from RRx-001 (PFS1) was 1.3 months. Since the initial data showed a low probability of tumour control during the induction RRx-001, 12 patients underwent CT scans after the first cycle and proceeded to platinum plus etoposide in case of tumour growth and were considered to have PD even if it did not meet the RECIST criteria for tumour progression in an attempt to decrease the probability of rapid clinical deterioration. The duration of benefit from each patient is shown in Fig. 4.

Since patients with platinum-resistant disease treated in the third line or beyond are the least likely to benefit from currently available treatment, we performed an exploratory analysis in this patient population. There were 14 patients, of whom 9 (64.2%) received subsequent platinum plus etoposide. Three of the 14 patients (21.4%) achieved PR and 4 (28.5%) had SD. The median PFS and OS were 5.8 months (95% CI 2.1–NR) and 8.6 months (95% CI 4.8–NR), respectively.

### Safety and tolerability

The most common treatment-emergent adverse events for RRx-001 included infusion site reaction (23%), decreased appetite (15.3%) and headache (11.5%), none considered drug related (Table 2). Infusion site reactions were characterised by a temporary discomfort at infusion site, paresthesias, pruritus or cough. There were four grade 3 or 4 toxicities, including decreased appetite, hypomagnesemia, hyperglycaemia and musculoskeletal pain. Four patients (15.3%) developed suspected tumour pseudoprogression during RRx-001 treatment, associated with pain and tumour size increase by scans, which were followed by improvement in symptoms and either tumour stabilisation or reduction with continued RRx-001 therapy.

**Table 2:** Treatment-emergent adverse events from RRx-001

Adverse event	Any grade	Grades 3 or 4
Infusion site pain	6 (23%)	0 (0%)
Decreased appetite	4 (15.3%)	1 (3.8%)
Headache	3 (11.5%)	0 (0%)
Fatigue	2 (7.6%)	0 (0%)
Constipation	2 (7.6%)	0 (0%)
Nausea	2 (7.6%)	0 (0%)
Insomnia	2 (7.6%)	0 (0%)
Rash	2 (7.6%)	0 (0%)
Musculoskeletal pain	2 (7.6%)	1 (3.8%)
Hypomagnesemia	2 (7.6%)	1 (3.8%)
Hyperglycaemia	2 (7.6%)	1 (3.8%)
Anaemia	2 (7.6%)	0 (0%)

Among patients treated with platinum plus etoposide, grade 1 and 2 adverse events (AEs) occurring in more than 5% of patients included nausea (61%), constipation (23%), diarrhoea (19%), abdominal pain (12%), vomiting (7.5%), stomatitis (8%), fatigue (31%), hypomagnesemia (8%), back pain (8%), musculoskeletal pain (12%), dyspnoea (12%), cough (7.5%), alopecia (8%) and thrombocytopenia (8%). Grade 3 and 4 AEs occurring in more than 5% of patients included anaemia (11.5%) and neutropenia (8%).

## Discussion

Our study showed that RRx-001 is well tolerated and associated with subsequent response to platinum plus etoposide in a previously treated SCLC patient population where benefit from systemic therapy is uncommon and of short duration. The results are particularly encouraging in patients with platinum-resistant disease treated in third-line or beyond, for whom the limited available data indicates that standard therapy has not been effective.

There is limited information on the efficacy of third-line systemic therapy for SCLC, with most data derived from retrospective studies. In a retrospective multicenter analysis including 120 patients with SCLC, the response rate to third-line chemotherapy was 18%, with median PFS and OS of 2.0 months and 4.7 months, respectively.^15^ Nevertheless, the patient population was very selected, with median PFS from first-line therapy of 9 months. Among the 41 patients with platinum-resistant disease, only 3 (7%) responded to third-line therapy. Similar results were observed in a larger retrospective study including 484 patients, with response rates, median PFS and OS of 15.1%, 2.0 months and 4.0 months, respectively.^16^ In a prospective study with the antibody−drug conjugate targeting trop-2 sacituzumab govitecan involving 50 patients, the ORR in patients platinum-resistant, and third-line and beyond were 16 and 17%, respectively.^17^

Rechallenge treatment with the original first-line platinum regimen has been studied mostly in patients with platinum-sensitive disease treated in the second-line setting. In a small study randomising 57 patients to second-line amrubicin or re-challenge with a platinum doublet in patients with platinum-sensitive disease, the ORR was 67% for amrubicin and 43% for re-challenge with platinum doublet, with median PFS of 5.4 and 5.1 months, respectively.^18^ Nevertheless, among the 11 patients with relapse between 3 and 6 months and treated with re-challenge platinum doublet, only three (27.2%) achieved response.

The ex-vivo method with the co-administration of RRx-001 with the patient’s own blood was introduced to mitigate the burning pain and vasodilation at the infusion site, which was observed in the phase 1 study, even in the lower dose cohorts, and caused by the displacement of nitric oxide (NO) from its binding site by RRx-001.^13^ Although the infusion site pain was not serious, it led to patient discomfort and the requirement of prolonged infusion times. Upon mixing RRx-001 with an aliquot of autologous blood, NO reenters the red blood cells prior to the infusion, resulting in a decreased direct contact with the vascular lumen. This method allowed RRx-001 to be infused in less than 1 h and with decreased pain at the administration site compared with the phase 1 study.

There are several possible mechanisms to explain the priming effect for RRx-001. One likely effect is the release of NO into the hypoxic tumour microenvironment. RRx-001 forms stable adducts with Cys-β^93^, which is the binding site for NO on haemoglobin. Binding of RRx-001 to Cys-β^93^ leads to NO displacement from haemoglobin. Although NO has been associated with tumour growth, through increasing vascular permeability, stimulating angiogenesis and invasiveness,^19,20^ it also induces apoptosis in tumour cells,^21,22^ particularly at higher levels.^23^ Chemotherapy resistance in hypoxic tumour cells can be attenuated by low concentrations of NO mimetics such as isosorbide dinitrate in an effect that is at least partly mediated by inhibition of the NFkB anti-apoptotic pathway.^24,25^

Another possible antitumor effect of RRx-001 is epigenetic modulation. In preclinical models, RRx-001 decreased the levels of DNA methyltransferases (DNMTs) in colon cancer and multiple myeloma cells.^26,27^ Furthermore, a study on murine squamous cell carcinoma of the head and neck (SCC VII) model showed decreased global 5-methylcytosine levels, decreased protein expression of DNMT1 and DNMT3, and increased acetylation of histones H3 and H4 after treatment with RRx-001, suggesting its role as an inhibitor of both DNMT and histone deacetylase (HDAC).^28^

In a phase I/II study involving 45 patients with previously treated NSCLC, treatment with subcutaneous azacytidine and the histone deacetylase (HDAC) inhibitor entinostat, the response rate was 4%.^29^ However, among the 19 patients that received subsequent therapy, four achieved major responses suggesting chemotherapy sensitisation. A similar finding was observed in the phase 1 study with RRx-001 where although only one of the 21 evaluable patients achieved PR (5%), four patients were subsequently re-challenged with previously effective chemotherapy regimens after progression on RRx-001 and achieved a prolonged survival, ranging from 10.0 months to 25.3 months.^13^

In our study, four patients (15.3%) developed pseudoprogression during the treatment with RRx-001. This phenomenon has been attributed to increased intratumoral inflammatory cell infiltrate or necrosis, occurs in approximately 4% of patients with solid tumours treated with immune checkpoint blockers, and prompted the development of the immune-related response criteria (irRC).^30,31^ Pseudoprogression was also observed in the phase 1 trial with RRx-001, where it was attributed to central necrosis, indicated by decreased density of pulmonary and non-pulmonary lesions, including hepatic and renal.^13^

Our study has several limitations, including a small sample size, absence of a control group and the lack of predictors for response to RRx-001. Furthermore, due to its exploratory nature, the sample size was not based on power considerations. Another limitation is the fact that seven patients had rapid tumour progression and did not receive the intended re-challenge with platinum plus etoposide. When the study was designed, the number of RRx-001 infusions required to re-sensitise the tumours to platinum plus etoposide was unclear. However, we observed responses to platinum re-challenge after ≤3 infusions of RRx-001, and with the rapid SCLC growth it became clear that the induction phase would have to be reduced to prevent rapid deterioration in some patients. Therefore, future studies will employ a short induction phase followed immediately by platinum plus etoposide.

Based on these encouraging initial results, a randomised phase 3 trial comparing RRx-001 followed by platinum plus etoposide to standard-of-care chemotherapy in previously treated patients with SCLC (REPLATINUM) has been initiated (NCT03699956).

## Supplementary information

Ethical Approval

## Data Availability

The data underlying the study are held by EpicentRx, Inc. the clinical trial sponsor.
