# Supplementary material for: RRx-001 followed by platinum plus etoposide in patients with previously treated small-cell lung cancer
Source: Br J Cancer. 2019 Jun 24;121(3):211–7. doi: 10.1038/s41416-019-0504-8 (PMC6738071; doi:10.1038/s41416-019-0504-8)
Supplement: Supplementary file 1 — Ethical Approval [file 41416_2019_504_MOESM1_ESM.docx]

**Ethical Approval and Consent to Participate**

| Institution Committee | Address |
| --- | --- |
| Walter Reed National Military Medical Center Institutional Review Board | 8901 Wisconsin Avenue  Bethesda, Maryland 20889-5600 |
| Memorial Hospital Institutional Review Board | 615 N. Michigan St.  South Bend, Indiana 46601 |
| Virginia Cancer Specialists, PC  Western Institutional Review Board (WIRB) | 8503 Arlington Blvd., Suite 400,  Fairfax, Virginia 22031 |
| Stanford University  Administrative Panels on Human Subjects in Medical Research | 300 Pasteur Drive  Stanford, California 94305 |
| Henry Ford Allegiance Health  Western Institutional Review Board (WIRB) | 1100 E. Michigan Ave., Suite 307,  Jackson, Michigan 49201 |
| West Virginia University  Advarra Institutional Review Board | 1 Medical Center Drive  Morgantown, West Virginia 26506 |
| The Washington University in St. Louis Institutional Review Board | 660 South Euclid Avenue, #8056  St. Louis, Missouri 63110 |
| University of Cincinnati  Western Institutional Review Board (WIRB) | 200 Albert Sabin Way, Suite 4002,  Cincinnati, Ohio 45267 |
| VA Connecticut Healthcare Systems Human Research Protection Program | 950 Campbell Avenue/111D  West Haven, Connecticut 06516 |
